# Functional Outcomes and Complications After Open Reduction and Internal Fixation of Mid-shaft Clavicle Fractures: A Retrospective Study

**DOI:** 10.7759/cureus.74302

**Published:** 2024-11-23

**Authors:** Muhammad A Hamid, Zubair Younis, Muhammad Mannan, Nayan Shrivastava, Rudra M Prabhu

**Affiliations:** 1 Trauma and Orthopedics, University Hospitals Birmingham NHS Foundation Trust, Birmingham, GBR; 2 Orthopedic Surgery, Bone and Joint Hospital, Srinagar, IND; 3 Trauma and Orthopedics, Royal Shrewsbury Hospital, Shrewsbury, GBR

**Keywords:** clavicle fractures, locking plates, orif, orthopedics, trauma

## Abstract

Introduction: Clavicle fractures are routinely encountered in orthopedic clinical practice and have often been the subject of debate when it comes to optimal treatment. Clavicle fracture surgery has come a long way with excellent pre-contoured superior locking plates available for fixation. This study aimed to evaluate a cohort of patients operated for displaced mid-shaft clavicle fractures by open reduction and internal fixation using superior clavicle locking plates.

Materials and methods: This is a retrospective cohort study of mid-shaft clavicle fracture patients who were operated on and had their fractures fixed using superior clavicle locking plates. We identified a total of 29 patients to be included in this study. The primary outcome measure was the quickDASH score at the time of discharge (12 weeks from surgery). Secondary outcome measures were the pattern and frequency of complications, and the need for metalwork removal.

Results: Our study had a male preponderance, with 19 (65.5%) patients being male. The most common mode of trauma was fracture secondary to a road traffic accident in 12 (41.4%) patients. All but one fracture united uneventfully. The mean quickDASH score was 0.94 at 12-week follow-up. Complications were noted in 12 (41.4%) patients, and the most common complication was hardware irritation in seven (24.1%) patients, followed by dysesthesia around the surgical scar in five (17.2%) patients.

Conclusion: Open reduction and internal fixation of the clavicle give a high likelihood of fracture union along with good shoulder function. In a particular cohort of patients, this offers a quick recovery and earlier return to activity. However, this must be balanced with the risk of complications in a considerable proportion of operated patients, some of which might necessitate a second surgery.

## Introduction

The clavicle is one of the most commonly fractured bones, accounting for 2.6-4% of all fractures [1]. The incidence of clavicle fractures in adults is 71 per 100,000 men and 30 per 100,000 women [1]. The incidence of fractures of the clavicle with age shows a bimodal distribution curve for males, with a peak incidence in young males less than 25 years old, and another peak in older males more than 55 years old. The distribution in females is unimodal, with a high incidence in older females, especially after 75 years of age [2].

Mid-shaft fractures of the clavicle account for approximately 75-80% of all clavicle fractures, and typically occur in younger persons. Distal third fractures represent about 15-25% of clavicle fractures. Medial third fractures are the least common, accounting for less than 5% of clavicle fractures [1]. Clavicle mid-shaft fractures are mostly treated non-operatively. This belief was reinforced by Neer's classic study in 1960, which reported a non-union rate of just 0.1% with non-operative treatment [3]. However, not all types of clavicle fractures are amenable to conservative treatment. Recent studies have shown that closed treatment of displaced middle-third fractures of the clavicle gives poorer overall outcomes [4,5]. Studies have also shown that operative treatment results in a lower rate of fracture non-union and improved patient-specific outcomes compared to non-operative treatment [6]. Despite the recent literature in support of operative fixation, a significant proportion of clavicle fractures can still be managed conservatively in a broad arm sling [7]. Surgery is warranted in mid-shaft clavicle fractures with significant displacement/shortening (>1.5 cm) or when the skin is at risk of compromise due to tenting. When opting to perform surgery, there are two most commonly employed techniques of fixation. These include internal fixation with intra-medullary devices or pre-contoured plates; both of which have predictable outcomes [8,9]. This study aimed to evaluate the functional outcomes and types of complications associated with the fixation of displaced mid-shaft clavicle fractures in adults with superior clavicle locking plates.

## Materials and methods

This was a retrospective cohort study conducted at the Bone and Joint Hospital, Srinagar, India, over a period of three years, from August 2017 to August 2020. Clavicle fractures (ICD-10 code S42.0) that were treated with open reduction and internal fixation using superior clavicle locking plates were included in the study. Patients were identified via the medical records register of our hospital, and their corresponding data and physical radiograph films were obtained from the medical records section.

Table 1 lists the inclusion and exclusion criteria we used for the purpose of this study. While we recognize some of the exclusion criteria mentioned are indications for surgery in a fractured clavicle, we have excluded them from this study as they would have confounded results [9].

**Table 1:** Inclusion and exclusion criteria.

Inclusion criteria	Exclusion criteria
All patients 16 years or older	Pathological fractures
Displacement >1.5 cm	Neuromuscular disabilities
Shortening >1.5 cm	Open fractures
Tenting of skin/skin at-risk	Previous surgery to the clavicle
-	Non-union

All our patients were operated on under general anesthetic, with the patient positioned in a beach-chair position. A bump was often placed behind the scapula to aid in reduction. An inferior incision was used over the clavicle to avoid the healed scar overlying the plate [10]. Following the incision of the platysma, the supraclavicular nerves were identified, and attempts were made to preserve them. The clavicle, being superficial, was easily accessible and the periosteum overlying the superior surface was cleared off to enable mobilizing fracture fragments. The implant used in all patients was a pre-contoured superior clavicle-locking compression plate. We routinely used a 3.5 mm or a 2.7 mm cortical screw to lag the often-found anterosuperior butterfly fragment wherever possible. Post-operatively, active use of the arm was encouraged, beginning with pendulum exercises and gradually moving on to a range of motion as pain would permit. We would normally limit weightlifting activities till six weeks following surgery until radiological signs of the bone union are observed on follow-up x-rays.

In addition to demographic details (age, sex), data pertaining to the mode of injury were obtained. The primary outcome measure was the patient-reported quickDASH score at 12 weeks post-surgery as obtained from their records. Secondary outcome measures included data pertaining to types of complications associated with surgery, and the need for metalwork removal.

## Results

A total of 29 patients were eligible for inclusion in our study. The mean age of the patients was 29.8±7.45 years. Males comprised 19 (65.5%) patients, and the most common mechanism of injury was road traffic accidents in 12 (41.3%) patients, followed by sports-related injuries in nine (31%) patients. These demographics are summarized in Table 2.

**Table 2:** Demographic details of the study cohort.

Variable	Value
Gender, n (%)	
Female	10 (34.5%)
Male	19 (65.5%)
Age, years (SD)	29.8 (7.45)
Mode of injury, n	
Road traffic accident	12 (41.4%)
Sports	9 (31%)
Falls	6 (20.7%)
Other	2 (6.9%)

**Figure 1:**
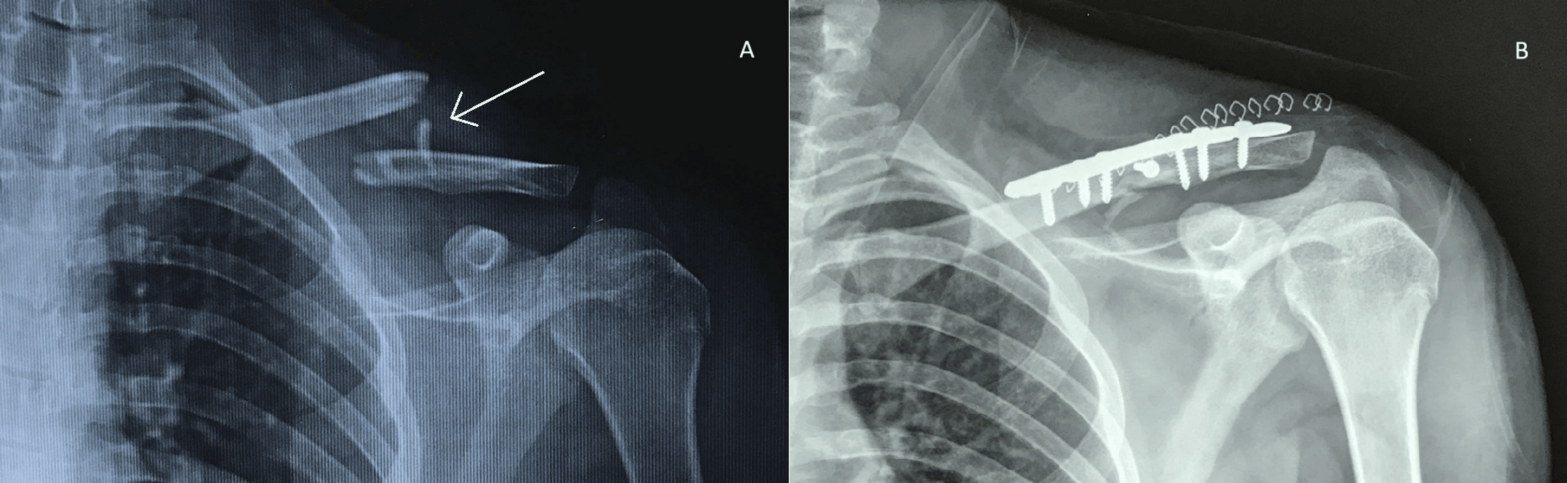
Pre- and post-operative x-rays of patient 17. (A) Pre-operative image with the often-seen anterosuperior butterfly fragment (arrow). (B) Post-operative image showing fixation construct.

The mean quickDASH score at two weeks, six weeks, and 12 weeks following surgery was 8.8, 2.1, and 0.94, respectively. We have depicted the pre- and post-operative x-rays of one of our operated patients in Figures 1A, 1B.

**Figure 2:**
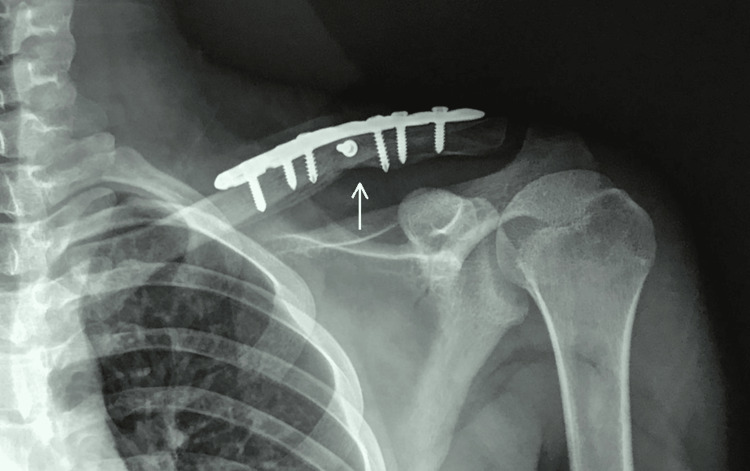
Final follow-up x-ray of patient 17. X-ray 12 weeks post-surgery demonstrating bony union (arrow).

The final follow-up x-ray for the same patient is depicted in Figure 2. A total of 12 (41.4%) patients suffered from complications related to the procedure. A summary of the complications encountered is outlined in Table 3.

**Table 3:** Summary of complications recorded in our patients.

Complication, n (%)	
Hardware irritation	7 (24.1%)
Dysesthesia	5 (17.2%)
Plate pullout	2 (6.9%)
Superficial infection	1 (3.4%)
Non-union	1 (3.4%)

**Figure 3:**
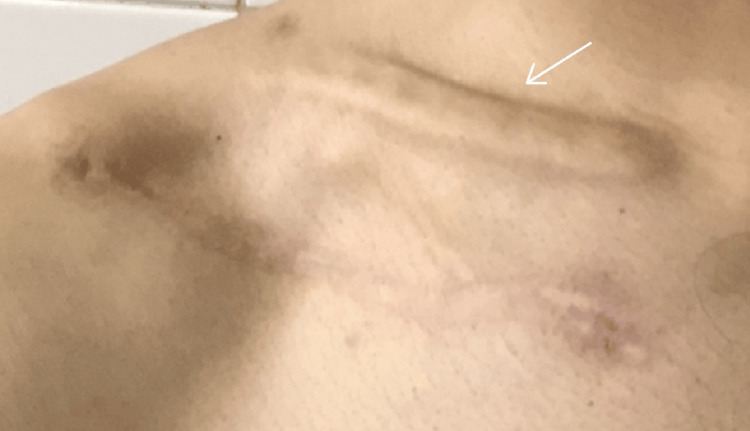
Implant prominence and irritation necessitating removal of metalwork. The inferiorly placed surgical incision heals well away from the underlying plate (arrow). However, this did not prevent the occurrence of hardware irritation in this patient.

Dysesthesias (loss of sensation/abnormal sensations) around the surgical scar were noted in five (17.2%) patients. Out of these five patients, symptoms improved in three over 12 weeks of follow-up, whereas in the other two, they did not. One (3.4%) patient suffered from a superficial stitch abscess six weeks following surgery. This was successfully evacuated during the clinic visit itself and managed with a short course of oral antibiotics. There were no stigmata of deep infection and the wound healed uneventfully. Two (6.9%) of our patients suffered from plate pullout over the lateral aspect of the clavicle noticed on their six-week follow-up x-rays. They were managed expectantly till fracture union and early implant removal were performed with satisfactory outcomes for both individuals. Hardware irritation was by far the most common complication in our series of patients, seen in seven subjects (24.1%). This took the form of either irritation (pain, discomfort) due to the plate being prominent, or irritation upon carrying sling bags/backpacks over the operated clavicle. Five of these patients opted for the removal of their hardware. Figure 3 depicts one such patient who was operated on for the removal of hardware due to implant irritation.

**Figure 4:**
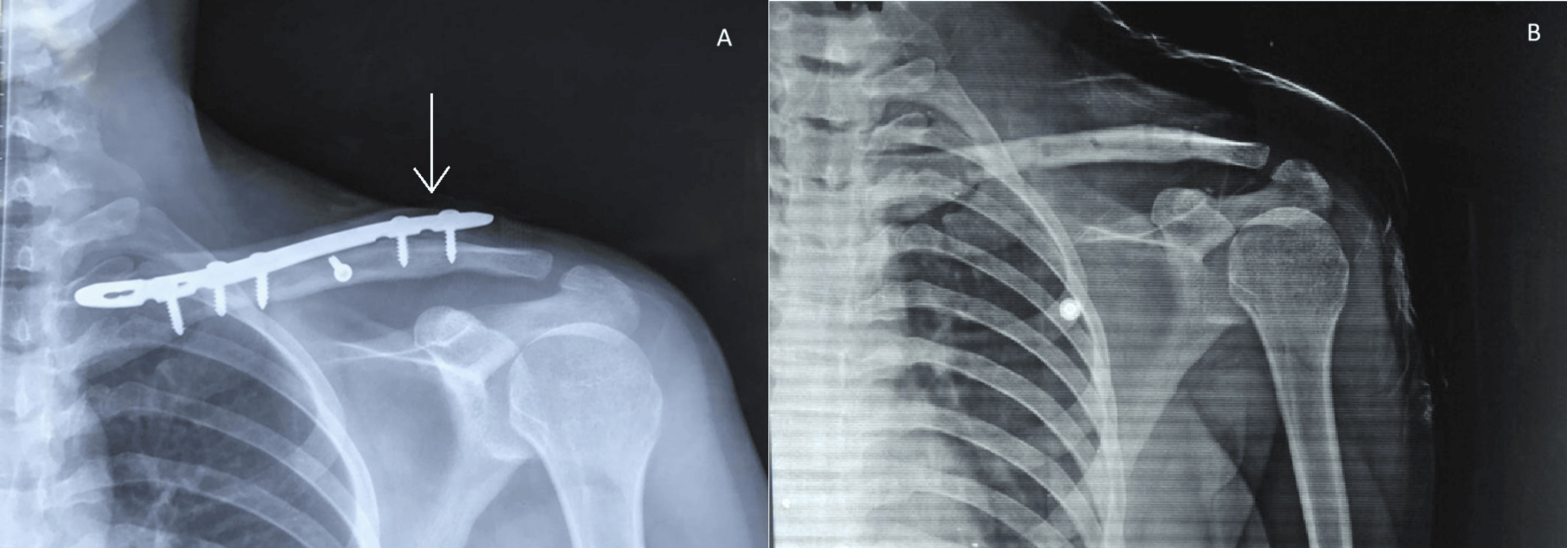
Plate pullout following fracture union necessitating removal of metalwork. (A) Nine months post-operative x-ray when the patient presented with complaints of hardware irritation (arrow depicting lateral pullout). (B) Post-operative x-ray following removal of metalwork.

**Figure 5:**
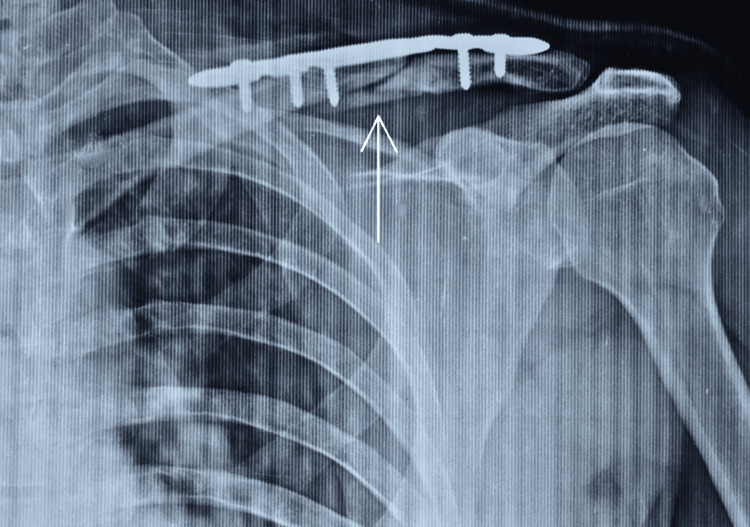
X-ray taken nine months post-operatively depicting absence of bridging callus (arrow).

Hardware removal was performed in a total of seven patients (24.1%). Five of these were purely for hardware irritation, whereas two plates were removed upon lateral pullout (Figures 4A, 4B). One (3.4%) of our patients developed a radiological non-union with the absence of bridging callus on his x-rays even at a nine-month follow-up (Figure 5). However, he was asymptomatic and was not keen on revision surgery. At the last follow-up, he was doing well with no symptoms.

## Discussion

Clavicle fractures are more commonly seen in young males, and that has been the pattern evident in our study as well. With a mean age of 29.8 (±7.45) years and 19 (65.5%) patients being male, our study mirrored what has been demonstrated in earlier research as well [11,12].

Among all the studies evaluated, road traffic accidents were by far the most common cause of clavicle fractures. In the present study, we also found that the most common cause of these injuries was road traffic accidents, accounting for 12 (41.4%) patients. The next common mechanism was a sports-related injury in nine (31%) patients, followed by falls in six (20.7%) patients. Some studies have demonstrated similar findings. We believe these discrepancies are usually the result of differing patient populations [13].

Fall from standing height was the next most common mechanism of injury in the studied patients, seen in six (20.7%) patients. Our findings were consistent with the findings of most other similar studies, where falls from standing height constituted the second largest group of patients who had sustained clavicle fractures [14,15]. We have summarized our comparison of the mode of trauma in Table 4.

**Table 4:** Mode of trauma compared in similar studies on clavicle fractures. RTA: road traffic accident

Study	Jiang and Qu [15]	Chalidis et al. [14]	Denise et al. [13]	Present study
RTA	48 (75%)	63 (53.2%)	24 (25%)	12 (41.4%)
Sports	6 (9.4%)	10 (7.2%)	23 (24%)	9 (31%)
Fall	10 (15.6%)	55 (39.6%)	6 (6%)	6 (20.7%)
Others	0 (0%)	0 (0%)	0 (0%)	2 (6.9%)

Complications

Twelve of our patients (41.4%) suffered from complications related to plate fixation. This high number is not entirely surprising, given that the ways to describe complications are variable and there is no standard for defining them when it comes to clavicle surgery. Most of the studies we compared did not report hardware irritation or dysesthesia as complications [16,17]. This "confounds" the complication rate given that those were two of the most common complications we noted in our study. A study comparing intra-medullary versus plate fixation in military servicemen reported a much higher incidence of complications in the plate fixation group. This was partly due to the inclusion of hardware irritation as a complication in seven (24%) patients, which contributed to an overall complication rate of nine (31%) patients [18]. Another study by Rongguang et al. reported hardware irritation in 19 (27.5%) patients operated with pre-contoured superior clavicle plates [19]. If we adjust for hardware irritation and paresthesia (i.e., discount them as complications), the overall complication rate in our study comes down to four (13.8%), which is comparable to most similar literature [16,20]. We feel, however, that this is an under-reporting of complications, and that hardware irritation and paresthesias/dysesthesias are certainly complications that should be studied in more detail.

The reasons for such a high incidence of hardware irritation might also be explained by the BMI of the patient population. There is some evidence to suggest that low BMI may be a factor contributing to the occurrence of hardware prominence and subsequent irritation following clavicle fracture surgery [19]. Unfortunately, we did not record the BMI of our patients but there is evidence to support the assumption that the BMI of high-income countries is higher than that of other countries (including India) [21]. This might explain the lower incidence of hardware irritation in those population groups [22,23]. We had one (3.4%) patient with a superficial wound infection that resolved uneventfully. This is similar to what others have described in the past [19,24].

In our study, two (6.9%) patients faced hardware failure, by means of a pull-out of the lateral end of the plate. This is similar to what has been reported previously as well [16,17,25]. This has been attributed to surgical technique in prior studies, especially improper choice and positioning of screws, which might have been the case in our patients as well [25]. However, we also seem to agree with the observations of Brouwer et al. in which a combination of superior surface plating, "ptosis" of the shoulder girdle, and shallow threads of the locking screws have been attributed as factors giving rise to a plate pullout [26]. Lastly, we observed one (3.4%) case who had a non-union. This was comparable to other studies conducted in the past [19,27].

Limitations

This study is limited by its sample size of only 29 patients. With this small sample, our observations might have limited external validity. Secondly, as this is a retrospective study, there was no element of randomization, and the patients who were operated on were the ones who chose this modality of treatment. The resulting bias might have been reflected by the high proportion of sports-related injuries in our study where surgical management was preferred by patients for earlier return to activity. Yet another limitation of this study is the absence of a control group; this limits our ability to extrapolate the incidence of complications we encountered in our patients to the wider population. Also, as our records did not capture data pertaining to the BMI of patients, this was a lost opportunity to study the correlation of BMI to the rate of hardware-related complications, as surmised by a few authors. As a considerable proportion of our operated patients reported hardware irritation, we would like this to be one of our focus areas for future research on clavicle fixations.

## Conclusions

Mid-shaft clavicle fractures managed by superior pre-contoured locking plate fixation behave predictably in terms of restoring anatomy and shoulder function. Surgery often affords a quick recovery back to baseline with a high union rate. However, the decision to undertake surgery must be balanced with the risk of implant-related complications. The ensuing hardware irritation might not affect shoulder function per se but is oftentimes reason enough to warrant second surgery for the removal of metalwork.

Lastly, research is lacking on prognosticating factors predicting the likelihood of complications like hardware irritation. There has been some research suggesting body weight might be one of them, but there is not enough evidence to support that assumption. More evidence is needed on the prognostication of these factors as that would help refine operative indications, making the decision-making process easier for the patient as well as the surgeon.
